# Predictive Factors and Clinical Impact of Radioactive Seed Migration After Prostate Brachytherapy: A Retrospective Study

**DOI:** 10.3390/curroncol32100567

**Published:** 2025-10-11

**Authors:** Shota Kikuchi, Takashi Fukagai, Jin Yamatoya, Kazuhiko Oshinomi, Masakazu Nagata, Masashi Morita, Kosuke Toyofuku, Atsuhito Sekimoto, Masako Kato, Madoka Morota, Yoshinori Ito

**Affiliations:** 1 Department of Urology, School of Medicine, Showa Medical University, Tokyo 142-8555, Japan; shota.93526@gmail.com (S.K.); jinjin@med.showa-u.ac.jp (J.Y.); oshikazu@med.showa-u.ac.jp (K.O.); nagatam@med.showa-u.ac.jp (M.N.); morita34@med.showa-u.ac.jp (M.M.); 2 Division of Radiation Oncology, Department of Radiology, School of Medicine, Showa Medical University, Tokyo 142-8555, Japan; k.toyofuku@med.showa-u.ac.jp (K.T.); a.sekimoto@med.showa-u.ac.jp (A.S.); mkrad@med.showa-u.ac.jp (M.K.); madmorot@med.showa-u.ac.jp (M.M.); yito@med.showa-u.ac.jp (Y.I.)

**Keywords:** brachytherapy, permanent seed implantation, prostate cancer, radiotherapy, seed migration

## Abstract

**Simple Summary:**

We evaluated the frequency and clinical impact of radioactive seed migration after prostate brachytherapy. Among 611 patients, 25% experienced seed migration, most commonly to the lungs, vasculature, or seminal vesicles. Although limited migration did not affect treatment outcomes, the migration of three or more seeds was associated with reduced dose coverage and with a trend toward worse biochemical control. Larger prostate volume, greater number of seeds, and more needles increased the risk of migration. Notably, patients who received neoadjuvant hormone therapy were less likely to experience seed migration. Seed migration is usually not harmful; however, understanding its risk factors is important to improve treatment planning. Using linked seeds and precise techniques may help minimize migration and optimize the effectiveness of brachytherapy in patients with prostate cancer.

**Abstract:**

Radioactive seed migration after low-dose-rate brachytherapy (LDR-BT) for prostate cancer is a known phenomenon; however, its clinical impact remains unclear. We retrospectively analyzed 611 patients treated with LDR-BT using loose iodine-125 seeds. Post-treatment imaging was used to assess seed migration. Treatment efficacy was evaluated using post-plan dosimetry (V100 and D90) and biochemical recurrence-free survival (bRFS). Seed migration was observed in 150 patients (24.5%) within 1–3 months post-treatment, involving a total of 210 seeds. Migration sites included lungs, vasculature, and seminal vesicles. Hematogenous migration was significantly associated with higher seed counts. Seminal vesicle migration was linked to increased needle usage and absence of neoadjuvant hormone therapy. No significant differences were observed in V100, D90, or bRFS between patients with or without seed migration. However, migration of ≥3 seeds correlated with significantly lower V100 and with a trend toward decreased bRFS. Limited seed migration appears to have minimal clinical impact. However, ≥3 migrated seeds may reduce dosimetric quality and affect treatment efficacy. Risk factors include larger prostate volume as well as higher seed and needle counts. Improved planning and using linked seeds may reduce migration and improve outcomes in LDR-BT for prostate cancer.

## 1. Introduction

Prostate cancer is the most common malignancy in men. Low-dose-rate brachytherapy (LDR-BT) using iodine-125 (I-125) seeds is the standard treatment option for localized prostate cancer, along with radical prostatectomy and external beam radiation therapy (EBRT). LDR-BT is characterized by shorter hospitalization periods, higher safety, and better preservation of quality of life post-treatment [1,2,3]. Recent series also confirm durable oncologic outcomes with I-125 LDR-BT [4]. From a value-based perspective, a 2025 systematic review reported that LDR-BT is often the most cost-effective option—particularly when oncologic outcomes are considered equivalent—compared to alternative modalities [5]. However, seed migration outside the prostate, where seeds were originally intended to remain, is a complication that occurs in 1.7–69.4% of cases [6,7], and may result in an insufficient radiation dose at the target site, thereby compromising therapeutic efficacy. Additionally, migrated seeds may cause adverse events at the sites where they relocate. The lungs are the most commonly reported destination for migrated seeds, and several studies have addressed this phenomenon. However, the migration of seeds is not limited to the lungs; they also migrate hematogenously into blood vessels within the heart and kidneys, wherein they drop out into the seminal vesicles [8,9,10,11]. Although pulmonary migration has been widely reported, comprehensive evaluations, including non-pulmonary and seminal vesicle migration, remain limited.

Therefore, we aimed to evaluate the location, timing, frequency and predictive factors of seed migration and the effect of the operator’s learning curve and its impact on treatment efficacy, based on a retrospective analysis of patients treated at our institution.

## 2. Materials and Methods

This retrospective study included 611 patients with prostate cancer who underwent LDR-BT using I-125 seeds, performed by a single surgeon (TF) between January 2005 and December 2021. Patients were excluded if post-treatment imaging studies (e.g., radiography or computed tomography [CT]) were unavailable or inadequate for evaluating seed migration. According to the treatment policy per the National Comprehensive Cancer Network (NCCN) guidelines, patients with a prostate volume exceeding 40 cc or an intermediate/high-risk classification received neoadjuvant hormone therapy, and some also underwent combined EBRT. In selected cases with prolonged waiting periods, short-course androgen deprivation therapy was administered to mitigate potential progression during scheduling delays. Neoadjuvant hormone therapy was administered for a median of 5 months (interquartile range [IQR] 4–8; mean 8.0; range 1–108 months). Treatment planning was conducted by the same team of radiation oncologists using real-time intraoperative planning with VariSeed software (Version 9.0, Varian Medical Systems, Palo Alto, CA, USA) [12]. The prescribed doses were 145 (2005–2009) and 160 Gy (post-2009) for monotherapy, and 110 Gy combined with 45 Gy EBRT. The institutional escalation to 160 Gy for monotherapy was adopted based on published evidence linking higher delivered doses (e.g., D90 thresholds) with improved biochemical control in I-125 LDR-BT [13]. A modified peripheral loading pattern was employed throughout the entire study period.

The loose seeds were implanted using a Mick applicator (Mick Radio-Nuclear Instruments, Bronx, NY, USA). Patient demographics and treatment-related data, including age, prostate specific antigen (PSA) level, Gleason score, T-stage, NCCN risk classification, pre-treatment prostate volume, use of hormone therapy, EBRT, number of seeds, number of needles, and post-plan V100 and D90, are listed in Table 1.

To evaluate the temporality of seed migration, only cases with imaging data available immediately postoperatively and 1 month or later were included. Images included chest and abdominal radiography, pelvic CT on the day after treatment, and pelvic CT post-planning at 1 month. In addition, all patients underwent repeat chest radiography and kidney-ureter-bladder (KUB) radiographs at 1–3 months postoperatively. Seed migration was defined as the detection of seeds outside the prostate. Migration to seminal vesicles was identified on CT as the presence of seeds in the vesicles at 1 day or 1 month post-treatment. In some cases, KUB radiographs were obtained 1 year after treatment.

To assess the influence of the surgeon’s learning curve, only cases performed by a single practitioner were included. We identified hematogenous migration to the lungs and other organs, dropout into seminal vesicles, and migration along the needle tract. In this study, we examined the effects of seed migration on therapeutic efficacy. Therefore, any deviation of the seeds from their planned positions was considered to have a potential clinical impact. As such, we included seed dropout into the seminal vesicles as a migration event because it represented a deviation from the intended plan.

Furthermore, we identified seven cases in which the seeds had migrated into the bladder through puncture needle tracts or moved perineally because of blood flow or negative pressure. However, these cases were excluded from the detailed analysis because, at our institution, intraoperative fluoroscopy was employed, and the operator was able to detect and compensate for these migrations by implanting additional seeds during the procedure. Thus, these factors were not considered to affect treatment efficacy.

Seed migration was confirmed based on imaging studies and categorized by location as follows: the lungs, other hematogenous sites, and seminal vesicles. To investigate potential risk factors, we assessed associations between seed migration and patient-related factors such as age, PSA, ISUP grade, pre-treatment prostate volume, and T stage (≥T3). Additionally, we examined treatment-related factors, including the total number of implanted seeds, number of needles, use of hormone therapy, and its combination with EBRT. The optimal cutoff values for continuous variables (e.g., prostate volume, total seed number, and number of needles) were determined using receiver operating characteristic (ROC) curve analysis. The cutoff point was chosen based on the maximum value of the Youden Index (sensitivity + specificity − 1), which identifies the threshold with the best trade-off between sensitivity and specificity. To evaluate the effect of the operator’s learning curve, cases were divided into early, middle, and late periods, and the migration incidence was compared across these groups.

Finally, to evaluate the clinical impact of seed migration on treatment outcomes, posttreatment dosimetric parameters (V100 and D90) and biochemical recurrence-free survival (bRFS) were analyzed. Univariate analyses were performed using the chi-square, t, and log-rank tests, whereas multivariate analyses were conducted using logistic regression. All statistical analyses were performed using JMP Pro 17.0 (SAS Institute Inc., Cary, NC, USA).

## 3. Results

### 3.1. Timing, Migration Sites, and Frequency of Seed Migration

Among the 611 patients evaluated, seed migration was confirmed in 89 cases (14.6%) on the day after treatment and in 150 cases (24.6%) on imaging obtained 1–3 months after treatment. Among the entire cohort, the number of migrated seeds ranged from 0 to 5 (median 0, mean 0.35, IQR 0–0). In patients with seed migration (*n* = 150), the number of migrated seeds ranged from 1 to 5 (median 1, mean 1.41, IQR 1–2). Among the 215 patients for whom imaging was available at 1 year, no new seed migrations were observed beyond those seen at 3 months, although in 3 cases, the previously identified seeds in the seminal vesicles disappeared. Based on these findings, 150 cases with confirmed seed migration within 1–3 months were included in the migration group.

A total of 41,280 seeds were implanted and 210 (0.5%) migrated. Migration sites included the seminal vesicles (*n* = 61, 10.0%), lungs (*n* = 39, 6.3%), and other suspected hematogenous routes, such as the pelvic vasculature (*n* = 64, 10.5%). Owing to overlapping cases, 92 patients (15.1%) exhibited migration to the lungs or vasculature.

### 3.2. Analysis of Predictive Factors for Seed Migration

As the mechanisms of hematogenous migration—including to the lungs—and dropout into the seminal vesicles differ, the cases were categorized into three groups for analysis: all cases with any seed migration (all groups), cases with hematogenous migration (lung/vascular group), and cases with seminal vesicle migration (SV group).

For each group, the presence or absence of seed migration was evaluated in relation to age, pre-treatment PSA (ng/mL), ISUP grade, clinical T stage (≥T3 vs. <T3), prostate volume at pre-plan (cc), number of needles used, total number of implanted seeds, use of neoadjuvant hormone therapy, and combination with EBRT. The results are summarized in Table 2.

In all groups, significant associations were observed between clinical T stage, prostate volume, seed count, needle count, hormone therapy, and EBRT. In the lung/vascular group, the prostate volume, seed count, hormone therapy, and EBRT were significantly associated. In the SV group, only hormone therapy was significantly associated with seed migration.

Based on these results, six factors—clinical T stage, prostate volume, seed count, needle count, hormone therapy, and EBRT—were selected for the univariate and multivariate logistic regression analyses. In the univariate analysis, clinical T stage did not reach statistical significance (*p* > 0.05); therefore, the multivariate logistic regression was conducted using the remaining five factors. For continuous variables, ROC curves were used to define cutoff values: prostate volume at 23 mL (area under the curve [AUC]: 0.59, sensitivity: 0.693, and specificity: 0.453), seed count at 68 (AUC: 0.616, sensitivity: 0.660, and specificity: 0.542), and needle count at 18 (AUC: 0.557, sensitivity: 0.390, and specificity: 0.753). Table 3 lists the results of the analysis.

In the multivariate analysis, total seed count (*p* = 0.0409) and needle count (*p* = 0.0371) were identified as significant predictors in all groups. In the lung/vascular group, the total seed count was significantly different (*p* = 0.0446). In the SV group, the univariate analysis showed significance for needle count and hormone therapy. In the multivariate analysis, needle count remained significant (*p* = 0.0102), and hormone therapy showed a trend toward significance (*p* = 0.052).

### 3.3. Relationship Between Seed Migration and the Learning Curve

To assess the effect of the operator’s learning curve, the cases were divided into early, middle, and late chronological periods, and the number of cases with confirmed seed migration was analyzed (Table 2). No significant changes were observed in the overall migration rate. However, in the lung/vascular group, a significant decreasing trend was observed over time: 20.6, 13.2, and 11.3% in the early, middle, and late periods, respectively (*p* = 0.0222).

### 3.4. Impact of Seed Migration on Treatment Efficacy

**Figure 1:**
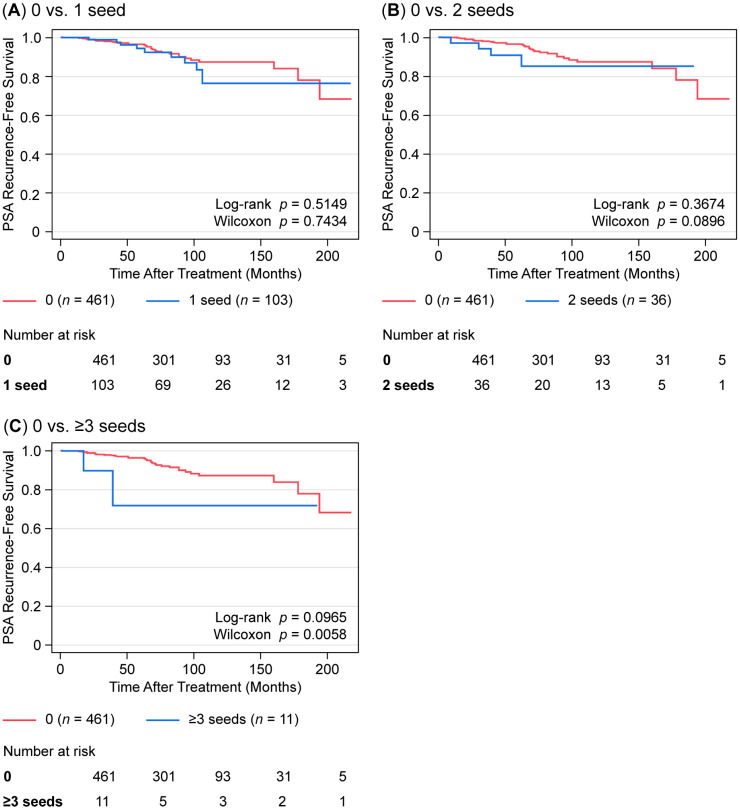
Biochemical recurrence-free survival according to the number of seed migrations. Panels compare 0 migrated seeds with (**A**) 1 seed, (**B**) 2 seeds, and (**C**) ≥3 seeds; cohort sizes are 0 (*n* = 461), 1 seed (*n* = 103), 2 seeds (*n* = 36), and ≥3 seeds (*n* = 11). Numbers at risk are shown below each panel. Within each panel, *p* values from the log-rank and Gehan–Breslow–Wilcoxon tests are reported.

To evaluate the effect of seed migration on treatment efficacy, bRFS and post-plan dosimetric parameters (V100 and D90) were analyzed. Biochemical recurrence was defined according to the Phoenix definition (PSA ≥ nadir + 2 ng/mL), and bRFS was assessed accordingly (Figure 1).

Comparison of V100 and D90 between the migratory and non-migratory groups showed no significant differences. When stratifying by the number of migrated seeds (1, 2, or ≥3), no significant differences in dosimetric parameters were found for 1 or 2 migrated seeds. However, in cases with ≥3 migrated seeds, a significantly lower V100 was observed (*p* = 0.0055, Table 4). As shown in Figure 1, the bRFS was not significantly different between patients with one migrating seed and those without migration. However, as the number of migrated seeds increased, a trend toward worse bRFS was observed.

## 4. Discussion

Numerous studies have addressed seed migration following LDR-BT for prostate cancer. However, the timing of assessment, migration sites, and evaluated endpoints varied across studies [6,7,14,15,16,17,18,19,20]. Regarding the timing of evaluation, our findings confirmed seed migration in 89 of 611 patients (14.6%) on the day after treatment and in 150 patients (24.6%) on imaging conducted 1–3 months post-treatment. Notably, 61 additional cases (10.0%) of migration were identified during the 1–3-month interval, beyond those observed on the day after treatment. Although previous studies reported only day 1 or 1–3 months post-treatment, Yamaguchi et al. reported similar findings in a study of 320 patients: 58 cases (18.1%) of migration on day 1, and an additional 31 cases identified one month later, totaling 89 cases (27.8%) [21]. No additional cases of migration were identified on imaging at 1 year, whereas Yamaguchi et al. reported three such cases. Maletzki et al. further reported seed migration even after 3 years [22]. Although rare, these findings indicate that seed migration can occur beyond 3 months. Because seed migration often occurs days to weeks after implantation, evaluation on day 1 alone was insufficient to capture all migration events. A post-treatment observation window of 1–3 months is more appropriate for assessing migration dynamics. Furthermore, as the radioactive activity of I-125 seeds significantly declines and becomes negligible by approximately 1 year post-implantation, migration assessments beyond this time frame are unlikely to yield clinically relevant information.

As mentioned previously, most studies have focused on hematogenous migration to the lungs. In our study, although 150 patients (24.6%) experienced seed migration, only 39 (6.3%) experienced seed migration to the lungs. The presumed mechanism of pulmonary migration involves a dense periprostatic venous plexus through which the seeds may enter the venous system and travel via the inferior vena cava and right heart to the pulmonary circulation. Seeds are thought to lodge in the peripheral pulmonary arterioles because of their size and rigidity [16]. The relatively low incidence of pulmonary migration in our cohort may be due to treatment planning strategies aimed at ensuring that all seeds are placed entirely within the prostate and minimizing entry into the periprostatic venous plexus. Nevertheless, we identified 64 cases (10.5%) of migration to other sites, including the bones, cardiovascular structures, kidneys, and retention within blood vessels. Of these, 53 (8.7%) involved migration to non-pulmonary vascular sites alone. Thus, limiting the evaluation of seed migration to the lungs alone likely underestimates its true extent.

Although studies have investigated the predictive factors for seed migration, few have distinguished between hematogenous migration and seminal vesicle dropout. In our study, univariate analysis revealed that the clinical T stage, prostate volume, number of needles used, total number of seeds, neoadjuvant hormone therapy, and EBRT were significantly associated with seed migration. Similarly, several studies have reported a higher incidence of seed migration with increased needle or seed counts [17,18,20], likely because of the higher absolute number of seeds. Our results are consistent with previous findings. Multivariate analysis revealed that the total seed count was the sole significant predictor of hematogenous migration, whereas the number of needles was a significant predictor of seminal vesicle migration. Dropout into seminal vesicles typically occurs when seeds are implanted near the base of the prostate, particularly on the rectal side. Thus, the number of needles placed near this region may be more relevant than the total seed count in predicting migration.

We also found that patients who underwent combined EBRT had significantly lower seed migration rates. This may be because the prescribed dose for combination therapy (110 Gy) was lower than that for monotherapy (145 or 160 Gy) and required fewer seeds. In our cohort, the average number of implanted seeds was 72.2 and 54.2 in the monotherapy and EBRT combination groups, respectively (*p* < 0.001), supporting this interpretation.

Prostate volume also contributes to increased migration, likely due to the need for more seeds and needle punctures in larger prostates. Our findings also indicate that patients who received neoadjuvant hormone therapy experienced significantly less seed migration. Nakano et al. suggested that hormone-induced prostate shrinkage might lead to increased seed clustering and migration [20]. Although it is common to reduce the number of seeds as the prostate shrinks, they did not compare prostate volumes between patients with and without hormone therapy. In their study, hormone therapy was administered to patients with very large glands (typically > 40 cc) to avoid pubic arch interference. Therefore, the treated prostates may have remained relatively large even after downsizing. In contrast, at our institution, hormone therapy was also used in intermediate- and high-risk patients for oncological reasons, indicating that not all hormone-treated patients initially had large prostates. In our study, hormone therapy significantly reduced prostate volume (24.6 cc with hormone therapy vs. 30.0 cc without hormone therapy; *p* < 0.001) and reduced the number of implanted seeds (63.2 vs. 74.1; *p* < 0.001), leading to fewer cases of migration. Additionally, hormone therapy may reduce the vascular caliber within and around the prostate, limiting seed mobility. Interestingly, dropouts into seminal vesicles occurred less frequently in patients who received hormone therapy. This may be explained by hormone-induced seminal vesicle atrophy [23], which narrows the luminal space and reduces the likelihood of seed entry.

Studies have also reported that increased operator experience correlates with decreased seed migration. Although all procedures in our study were performed by a single operator, we found no significant temporal trends in the overall migration rates. However, hematogenous migration decreased over time, suggesting that this improved technique may reduce inadvertent vascular implantation. Even after more than 500 cases, migration into the blood vessels remained at approximately 10%. This suggests that vascular migration was unavoidable in the presence of free seeds. To mitigate this, more precise identification of the vascular anatomy using advanced ultrasound or linked seeds should be considered.

Although some studies have reported the impact of seed migration on treatment efficacy, most reported no significant changes in V100 or D90 [17,18]. Gao et al. argued that seed migration theoretically reduces radiation coverage [24]. Nakano et al. also reported significantly lower D90 and V100 values in patients with ≥2 migrated seeds [20]. Our findings are consistent with these studies; no significant impact on V100 was observed with up to 2 migrated seeds, but cases with ≥ 3 showed significantly lower V100 (*p* = 0.0055). Few studies have examined the effect of seed migration on clinical outcomes. Stone et al. reported no significant difference in recurrence rates over 9 years in patients with and without seed migration [7]. Similarly, in our study, no significant difference in PSA-based biochemical recurrence-free survival was observed. However, a trend toward poorer outcomes was noted in patients with ≥ 3 migrated seeds (log-rank test, *p* = 0.0965; Wilcoxon test, *p* = 0.0058). These findings suggest that up to 2 migrated seeds may not significantly affect outcomes, but more than 3 seeds may warrant closer follow-up.

Despite the strengths of this study, it has limitations. First, the timing of seed migration evaluation ranged from 1 to 3 months, and this variability may have influenced the detection of migration. Second, although seed migration is virtually nonexistent with linked seeds, we exclusively investigated free (loose) seeds. Previous studies have consistently shown that linked seeds result in significantly lower migration rates [7,25]. Our findings revealed that the frequency of seed migration (14.6% of patients/0.5% of implanted seeds) is specific to cohorts using loose seeds with the Mick applicator technique; thus, our findings may not be generalizable to all seed types, and further comparative studies are warranted. Third, although migration of ≥3 seeds was associated with lower D90/V100, we did not conduct spatial mapping of intraprostatic failures to pre-migration dose distributions. Given that migration was mainly identified on radiographs and that post-migration pelvic CT was not uniformly acquired, we could not determine whether local relapse originated from below-dose (cold-spot) regions created by seed loss. Accordingly, this mechanism remains hypothesis-generating and warrants prospective evaluation with standardized post-migration imaging and, when feasible, image-guided biopsy or prostate-specific membrane antigen positron emission tomography/CT. Fourth, although we assessed the incidence and risk factors for seed migration, we did not evaluate the clinical consequences of migrated seeds. Documented cases of complications arising from seeds that migrate to the lungs or cardiovascular system are available [26,27,28]. Future studies should examine the potential effects of hematogenous migration on these organs.

## 5. Conclusions

In this study, we identified several factors significantly associated with seed migration following prostate brachytherapy using loose seeds. A larger prostate volume, increased seed number, and more needles were positively associated with the migration risk, whereas hormone therapy appeared to reduce it. These results suggest that careful consideration of the prostate size, seed planning, and procedural techniques is essential to minimize seed migration. Future research should address the clinical impact of migrated seeds and evaluate the role of alternative seed types, such as linked seeds, in reducing migration.

## Figures and Tables

**Table 1:** Patient characteristics (*n* = 611).

Variables	Value (Range or %)
Age (years), median (range)	71 (47–86)
Initial PSA (ng/mL), median (range)	7.46 (0.114–391)
Gleason score (ISUP grade)	
Gleason ≤ 6 (ISUP 1)	258 (42.4)
Gleason 7 (3 + 4) (ISUP 2)	140 (23.0)
Gleason 7 (4 + 3) (ISUP 3)	96 (15.8)
Gleason 8 (4 + 4, 3 + 5, 5 + 3) (ISUP 4)	71 (11.7)
Gleason 9–10 (ISUP 5)	43 (7.1)
Unknown	3
Clinical T stage	
<T3	567 (93.9)
≥T3	37 (6.1)
Unknown	7
NCCN risk classification	
Low	203 (33.3)
Intermediate	252 (41.4)
High	154 (25.9)
Unknown	2
Prostate volume (cc), median (range)	24.64 (8.1–70.5)
Number of seeds, median (range)	68 (25–103)
Number of needles, median (range)	16 (10–31)
Treatment method: Hormone therapy	
Yes	370 (60.6)
No	241 (39.4)
Treatment method: Treated with EBRT	
Yes	156 (25.5)
No	455 (74.5)
V100 (%), median (range)	95.3 (66.0–99.9)
D90 (Gy), median (range) Combination	130.2 (78.9–193.4)
Mono	176.1 (91–225.3)

PSA, prostate specific antigen; EBRT, external beam radiation therapy; ISUP, International Society of Urological Pathology; NCCN, National Comprehensive Cancer Network.

**Table 2:** Seed migration rate according to site.

	All Sites (*n* = 611)			Lung, Vein, and Other Organs (*n* = 611)			Seminal Vesicle (*n* = 611)		
Variables	Migration (*n* = 150)	No Migration (*n* = 461)	*p* Value	Migration (*n* = 92)	No Migration (*n* = 519)	*p* Value	Migration (*n* = 61)	No Migration (*n* = 550)	*p* Value
Age (years), median (range)	71 (51–84)	71 (47–86)	0.4825	71 (51–84)	71 (47–86)	0.5359	71 (55–84)	71 (47–86)	0.8448
Initial PSA (ng/mL), median (range)	7.18 (4–75.4)	7.50 (0.114–391)	0.1529	6.86 (4–75.4)	7.52 (0.11–391)	0.2548	7.5 (4.3–56.6)	7.45 (0.11–391)	0.2548
ISUP grade, *n* (%)									
1	65 (25.2)	193 (74.8)	0.1574	43 (16.7)	215 (83.3)	0.4410	23 (8.9)	235 (91.1)	0.1278
2	42 (30.0)	98 (70.0)		25 (17.9)	115 (82.1)		18 (12.9)	122 (87.1)	
3	23 (24.0)	73 (76.0)		12 (12.5)	84 (87.5)		12 (12.5)	84 (87.5)	
4	15 (21.1)	56 (78.9)		7 (9.9)	64 (90.1)		8 (11.3)	63 (88.7)	
5	5 (11.6)	38 (88.4)		5 (11.6)	38 (88.4)		0 (0)	43 (100)	
T stage, *n* (%)									
<T3	144 (25.4)	423 (74.6)	0.0456 *	89 (15.7)	478 (84.3)	0.090	60 (10.6)	507 (89.4)	0.1233
≥T3	4 (10.8)	33 (89.2)		2 (5.4)	35 (94.6)		1 (2.7)	36 (97.3)	
NCCN risk classification, *n* (%)									
Low	54 (26.6)	149 (73.4)	0.0599	38 (18.7)	165 (81.3)	0.060	18 (8.9)	185 (91.1)	0.1557
Intermediate	69 (27.4)	183 (72.6)		39 (15.5)	213 (84.5)		32 (12.7)	220 (87.3)	
High	27 (17.5)	127 (82.5)		15 (9.7)	139 (90.3)		11 (7.1)	143 (92.9)	
Prostate volume (cc), median (range)	27.9 (10.5–70.5)	24.2 (8.1–60.4)	0.0004 *	29.35 (10.5–70.5)	24.2 (8.1–60.4)	0.0001 *	24.1 (11.74–49.9)	24.69 (8.1–70.5)	0.7428
Number of seeds, median (range)	70.5 (40–90)	66 (25–85)	0.0001 *	75 (40–103)	66 (25–100)	0.0001 *	69 (42–97)	67 (25–103)	0.2426
Number of needles, median (range)	17 (10–31)	16 (10–27)	0.0181 *	17 (10–31)	16 (10–27)	0.0604	17 (11–22)	16 (10–31)	0.1433
Hormone therapy, *n* (%)									
Yes	74 (20.0)	296 (80.0)	0.0012 *	45 (18.7)	196 (81.3)	0.0438 *	28 (7.6)	348 (92.4)	0.0136 *
No	76 (31.5)	165 (68.5)		47 (12.7)	323 (87.3)		33 (13.7)	208 (86.3)	
Combination with EBRT, *n* (%)									
Yes	26 (16.7)	130 (83.3)	0.008 *	13 (8.3)	143 (91.7)	0.0065 *	11 (7.1)	145 (92.9)	0.1568
No	124 (27.3)	331 (72.7)		79 (17.4)	376 (82.6)		50 (11.0)	405 (89.0)	
Order of patients treated, *n* (%)									
0–204 (204)	61 (29.9)	143 (70.1)	0.0533	42 (20.6)	162 (79.4)	0.0222 *	21 (10.3)	183 (89.7)	0.1330
205–408 (204)	40 (19.6)	164 (80.4)		27 (13.2)	177 (86.8)		14 (6.9)	190 (93.1)	
408–611 (203)	49 (24.1)	154 (75.9)		23 (11.3)	180 (88.7)		26 (12.8)	177 (87.2)	

* Statistically significant difference between groups (*p* < 0.05). Continuous variables were compared using the t-test; categorical variables with the chi-square test. *p* values for categorical variables refer to the overall comparison across categories.

**Table 3:** Predictive factors for migration according to univariate and multivariate analyses.

Variables (Reference/Comparator)	Univariate OR (95% CI)	*p* Value	Multivariable aOR (95% CI)	*p* Value
A. Predictors of all sites migration (*n* = 611)				
Prostate volume (cc) (≥23 vs. <23)	1.72 (1.16–2.54)	0.007 *	0.92 (0.55–1.54)	0.7403
Number of seeds (≥68 vs. <68)	2.30 (1.57–3.38)	<0.0001 *	1.75 (1.02–3.00)	0.0409 *
Number of needles (≥18 vs. <18)	1.97 (1.34–2.91)	0.0006 *	1.56 (1.03–2.37)	0.0371 *
Clinical T stage (≥T3 vs. <T3)	0.35 (0.12–1.02)	0.055	-	-
Hormone therapy (Yes vs. No)	0.54 (0.37–0.79)	0.0013 *	0.71 (0.47–1.06)	0.091
Combination with EBRT (Yes vs. No)	0.53 (0.33–0.85)	0.0087 *	0.80 (0.47–1.35)	0.3963
B. Predictors of lung/vein/other organ migration (*n* = 611)				
Prostate volume (cc) (≥23 vs. <23)	2.40 (1.44–3.99)	0.0008 *	1.38 (0.72–2.62)	0.332
Number of seeds (≥68 vs. <68)	2.86 (1.76–4.65)	<0.0001 *	1.96 (1.01–3.80)	0.0446 *
Number of needles (≥18 vs. <18)	1.60 (1.00–2.55)	0.0472 *	1.13 (0.69–1.85)	0.6407
Clinical T stage (≥T3 vs. <T3)	0.31 (0.07–1.29)	0.1086	-	-
Hormone therapy (Yes vs. No)	0.63 (0.40–0.98)	0.0449 *	0.92 (0.57–1.49)	0.7422
Combination with EBRT (Yes vs. No)	0.43 (0.23–0.80)	0.0078 *	0.68 (0.34–1.35)	0.2712
C. Predictors of seminal vesicle migration (*n* = 611)				
Prostate volume (cc) (≥23 vs. <23)	1.12 (0.65–1.93)	0.6878	0.67 (0.32–1.37)	0.2719
Number of seeds (≥68 vs. <68)	1.45 (0.85–2.48)	0.1746	1.08 (0.50–2.32)	0.8402
Number of needles (≥18 vs. <18)	2.20 (1.28–3.79)	0.0042 *	2.15 (1.20–3.87)	0.0102 *
Clinical T stage (≥T3 vs. <T3)	0.23 (0.03–1.74)	0.1565	-	-
Hormone therapy (Yes vs. No)	0.52 (0.30–0.88)	0.0148 *	0.56 (0.32–1.00)	0.052
Combination with EBRT (Yes vs. No)	0.61 (0.31–1.21)	0.1603	0.74 (0.35–1.58)	0.4444

Data are shown as odds ratios (OR) with 95% confidence intervals (CI). Univariate and multivariable logistic regression models were fitted for each outcome. Reference categories are indicated in parentheses in the Variables column. Asterisks (*) denote *p* < 0.05 as reported.

**Table 4:** Change of V100 and D90 according to the number of seed migrations.

Number of Migrated Seeds (*n*)	V100 (Median (Range))	*p* Value (vs. No Migration)	D90 (Median (Range))	*p* Value (vs. No Migration)
No migration (*n* = 461)	95.428 (66.862–99.943)	-	163.106 (78.856–225.253)	-
1 seed (*n* = 103)	95.307 (67.486–99.73)	0.9323	171.998 (89.622–202.625)	0.055
2 seeds (*n* = 36)	94.82 (65.987–99.043)	0.4542	172.594 (101.441–197.625)	0.2835
≥3 seeds (*n* = 11)	90.126 (76.402–95.786)	0.0055 *	144.206 (110.345–179.231)	0.1343

* Significant factors for predicting migration (*p* < 0.05).

## Data Availability

The data that support the findings of this study are available from the corresponding author upon reasonable request.

## Abbreviations

The following abbreviations are used in this manuscript:

LDR-BT	Low-dose-rate brachytherapy
EBRT	External beam radiation therapy
CT	Computed tomography
NCCN	National Comprehensive Cancer Network
PSA	Prostate specific antigen
ROC	Receiver operating characteristic
AUC	Area under the curve
